# Unrecognized Pseudohypoparathyroidism Type 1A as a Cause of Hypocalcemia and Seizures in a 64-Year-Old Woman

**DOI:** 10.1155/2019/8456239

**Published:** 2019-01-09

**Authors:** Patrizia Del Monte, Carla Micaela Cuttica, Alessandro Marugo, Luca Foppiani, Daniela Audenino, Tomasz Tadeusz Godowicz, Francesca Marta Elli, Giovanna Mantovani, Emilio Di Maria

**Affiliations:** ^1^Endocrine Unit, Galliera Hospital, Genoa, Italy; ^2^Internal Medicine Unit, Galliera Hospital, Genoa, Italy; ^3^Neurology Unit, Galliera Hospital, Genoa, Italy; ^4^Neurosurgery Unit, Galliera Hospital, Genoa, Italy; ^5^Fondazione IRCCS Ca' Granda Ospedale Maggiore Policlinico, Endocrinology and Diabetology Unit, Department of Clinical Sciences and Community Health, University of Milan, Milan, Italy; ^6^Department of Health Sciences, University of Genoa; Medical Genetics Unit, Galliera Hospital, Genoa, Italy

## Abstract

Pseudohypoparathyroidism type 1A (PHP1A) is usually diagnosed in childhood or early adulthood. We describe the case of a 64-year-old woman admitted to the Neurological Unit for recurrent episodes of loss of consciousness and seizures. Glycemia and ECG were normal, while hypocalcemia was noted. Clinical history revealed carpo-pedal spasm since the age of 30 years, cognitive impairment, hypothyroidism since early adulthood, and menopause at 30 years. She was taking oral calcium and cholecalciferol for chronic hypocalcemia. Physical features suggested Albright's osteodystrophy. Blood calcium was confirmed low, with increased parathyroid hormone, moderate 25OH-vitamin D deficiency, and normal creatinine. Brain CT scan revealed calcifications of the basal ganglia, cortical and subcortical white matter, and cerebellum. Therapy was switched to oral calcitriol, with normalization of calcium levels; levetiracetam was started and no further seizures occurred. The clinical diagnosis of PHP1A was confirmed by molecular analysis, which demonstrated the heterozygous c.568_571del mutation of the* GNAS* gene. Our report illustrates the natural history of a patient with PHP1A, which went undiagnosed until the age of 64 years, with multi-hormonal resistance and clinical sequelae evolving throughout life, and underlines the importance of diagnosing this rare disease, which has a great impact on patients and their family life.

## 1. Introduction

Pseudohypoparathyroidism (PHP) is a rare congenital disorder, its estimated prevalence being 0.34 - 1.1 in 100.000; it is characterized by impairment of the parathyroid hormone (PTH) signaling pathway, with target organ resistance to the action of PTH [1, 2]. The term PHP comprises several related and highly heterogeneous diseases with genetic/epigenetic causes [3, 4]. PTH exerts its actions by binding to a transmembrane G-protein-coupled receptor, activating cAMP formation. The classical form of PHP, pseudohypoparathyroidism type 1A (PHP1A), which is associated with Albright's hereditary osteodystrophy (AHO), is caused by* de novo* or autosomal dominantly inherited inactivating mutations within the G_s*α*_-coding* GNAS* gene (20q13.3; OMIM #139320) [5]. The maternal allele is the predominant source of G_s*α*_-expression in the proximal renal tubule, thyroid, pituitary, and gonads, since paternal G_s*α*_ expression is silenced in these tissues; in other tissues, however, G_s*α*_ expression is biallelic. Therefore, an inactivating G_s*α*_ mutation causes PHP1A (AHO clinical features and hormone resistance) in the case of maternal inheritance, while the same mutation on the paternal allele results in AHO clinical features only, a condition known as pseudo-pseudo-hypoparathyroidism (PPHP).

PHP1A patients often develop target-organ resistance to other hormones that act through G_s*α*_-coupled receptors, particularly TSH; resistance to gonadotrophins and GHRH is also reported.

The disease is usually diagnosed in childhood or early adulthood age, and few studies have addressed its natural history, in which its features evolve during the subject's lifetime. Moreover, to our knowledge, no diagnoses have been made after 60 years of age in patients not studied for known familial disease.

## 2. Clinical Case

A 64-year-old woman was admitted to the Neurological Unit of our hospital for recent recurrent episodes of loss of consciousness and seizures. Glycemia and ECG were normal, while hypocalcemia was present.

She has a normal brother and both her parents died in old age; her mother had cognitive impairment. Her clinical history evidenced carpo-pedal spasm since the age of 30 years, cognitive impairment, hypothyroidism diagnosed in early adulthood, spontaneous menarche, and oligomenorrhea followed by amenorrhea at the age of 30, which was diagnosed as precocious menopause. She was unmarried and had no pregnancy. She underwent bilateral hip arthroprosthesis at 45 and 50 years of age.

She was taking oral calcium (600 mg daily) and cholecalciferol (400 IU daily) for chronic hypocalcemia, diagnosed about 30 years earlier. She was also on therapy with perindopril for hypertension, atorvastatin for hypercholesterolemia, and L-thyroxine.

Physical examination revealed short stature (145 cm), slight overweight: 52 Kg (BMI: 25 Kg/m^2^), round facies, enlarged base of the nose, and brachydactyly. Her blood chemistry evidenced hypocalcemia (7.7 mg/dl, n.v. 8.2-10.2) with increased PTH levels (169 pg/ml, n.v. 15-65 pg/ml, intact PTH immunoassay), moderate 25OH vitamin D deficiency (22 ng/ml; n.v. ≥ 30), normal creatinine (1 mg/dl), and albumin (3.9 g/dl).

**Figure 1:**
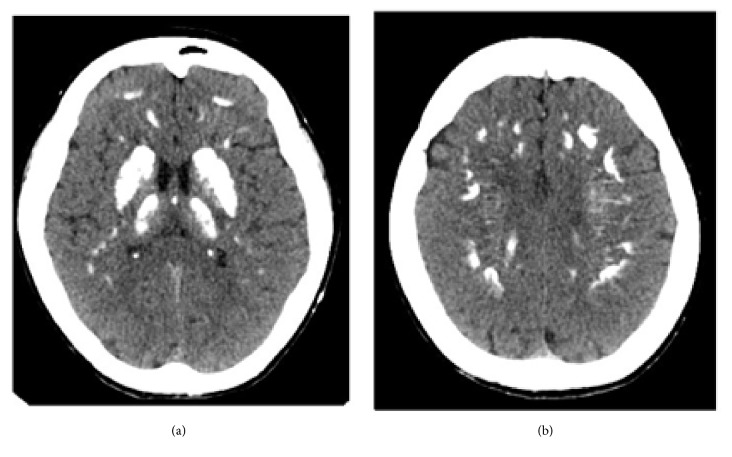
Basal ganglia (a) and subcortical (b) calcifications (CT scan).

**Figure 2:**
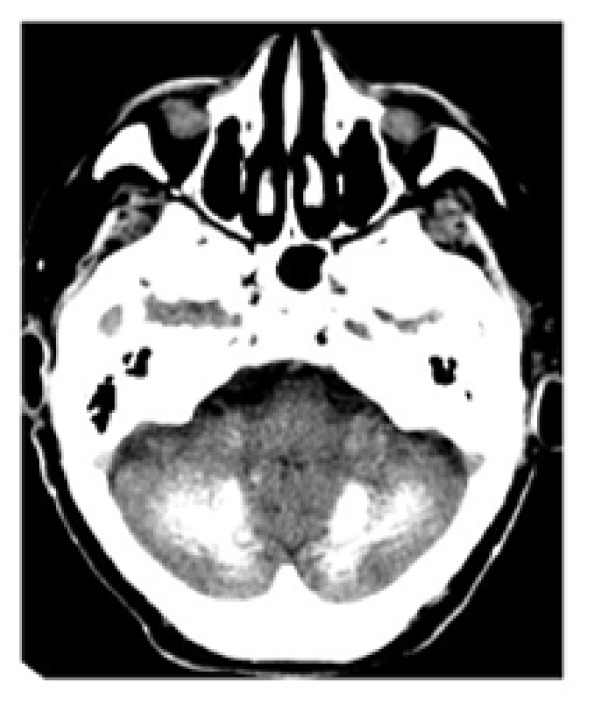
Cerebellar calcifications (CT scan).

**Figure 3:**
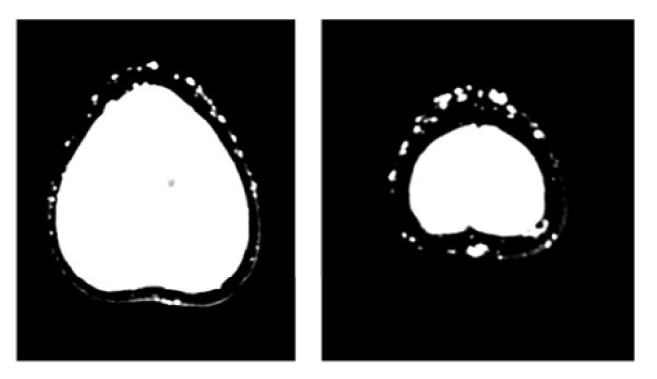
Extracranial subcutaneous ectopic calcifications (CT scan).

Brain computed tomography (CT) revealed calcifications of the basal ganglia, the cortical and subcortical white matter, and the cerebellum (dentate nuclei); subcutaneous pericranial ectopic calcifications were also present (Figures 1, 2, and 3). Hand radiography confirmed shortness of the metacarpal bones and scapho-trapezoidal fusion (not shown). Bone mineral density of the spine and femoral bone was normal. Abdominal ultrasonography did not reveal kidney stones. The clinical picture was suggestive of PHP1A. The patient was therefore promptly switched to 1,25 (OH)_2_ vitamin D (calcitriol 0.75 mcg daily, in 3 split doses) associated with oral calcium supplementation (1000 mg daily in 2 split doses, after lunch and dinner), with normalization of calcemia (9.2 mg/dl) and a decrease in PTH level (36 pg/ml). Serum phosphate was normal on therapy (4.3 mg/dl), while 24-hour urinary calcium on therapy was above the normal range (321 mg/24 h, n.v. <4 mg/kg/body weight); we do not have a clear explanation for this, since urinary calcium excretion is not normally increased in this condition. However, a laboratory assay pitfall or an incorrect urine collection by the patient cannot be ruled out. A further urinary calcium control one month later, however, proved normal (200 mg/24 h). In addition, therapy with levetiracetam (1000 mg daily in 2 split doses) was started, and no further seizures or muscle spasms occurred.

The patient and her family underwent a genetic counseling session and gave informed consent to genetic analysis. Mutation analysis of the* GNAS* gene was performed on DNA extracted from a blood sample by means of Sanger sequencing and MLPA according to standard methods. The analyses revealed a heterozygous c.568_571del (p.Asp190Metfs*∗*13) frameshift mutation. The combination of physical, biochemical, and genetic findings led to the diagnosis of PHP1A.

FT4 and TSH levels were normal during substitutive treatment with L-tiroxine (75 mcg daily). Anti-thyroperoxidase and anti-thyroglobulin antibodies were negative and thyroid ultrasonography was normal, suggesting that TSH resistance was the cause of her hypothyroidism.

LH and FSH levels were in the menopausal range (FSH: 47 mU/ml, LH: 26 mU/ml); the history of premature menopause, however, was suggestive of the development of progressive LH and FSH resistance. Prolactin, basal cortisol, and ACTH levels were in the normal range. Basal growth hormone was 0.63 ng/ml, with IGF-1 levels (129 ng/ml) in the normal range for age. Calcitonin levels were increased (range: 44-92 pg/ml, n.v. <10 pg/ml) (she was not taking any proton-pump inhibitor), while carcinoembryonic antigen (CEA) was normal. Abdominal ultrasonography was normal, as were chest-X-ray and mammography. Our interpretation is that calcitonin resistance was also present, as part of the patient's multi-hormonal resistance due to impairment of the cAMP activation pathway [6, 7].

Six months later, during follow-up evaluation, the patient's calcium level was seen to have increased to 10.6 mg/dl and PTH had decreased to 32 pg/ml; oral calcium supplementation was gradually reduced and then discontinued, and calcitriol was reduced to 0.25 mcg twice daily; subsequent controls revealed normal calcium (10 mg/dl) and slightly increased PTH (72 pg/ml) levels. She had no further loss of consciousness or seizures; on neurological examinations, levetiracetam treatment was confirmed, also on account of the frailty of the patient.

## 3. Discussion

This is an unusual case of the first diagnosis of PHP type 1 A in a 64-year-old woman, who presented with hypocalcemia and recent recurrent episodes of loss of consciousness and seizures. Combined evaluation of her clinical history, physical features, blood chemistry, and radiological findings suggested this diagnosis. A retrospective interpretation of her history showed how the disease had evolved during the patient's lifetime: in early adulthood, she developed PTH resistance-related hypocalcemia, carpo-pedal spasm, and hypothyroidism due to TSH resistance; around the age of 30 years, she suffered ovarian failure due to FSH and LH resistance; this was followed by bilateral hip disease requiring arthroprosthesis at 45 and 50 years and worsening hypocalcemia, which was unresponsive to cholecalciferol and oral calcium supplementation; finally, recurrent seizures recently ensued. Calcitonin resistance was also present, while IGF-1 was normal. She had no kidney stones; this is in keeping with the observation that urinary calcium levels are not markedly increased in these patients, as the action of PTH is maintained in the distal renal tubule, where GNAS gene expression is biallelic. Switching to calcitriol treatment promptly corrected the woman's hypocalcemia, and she had neither muscle spasms nor seizures on anti-epileptic medication. Both hypocalcemia and the development of multiple cortical and subcortical calcifications could have contributed to the development of seizures.

Based on the patient's clinical characterization, the targeted mutation analysis of the* GNAS* gene identified a known mutation already associated with PHP1A [8], thus allowing the clinical diagnosis to be confirmed. The mutation was presumably inherited from the mother, who was referred to as having cognitive impairment. However, as no sample was available, this hypothesis was not formally confirmed.

In accordance with the recommendations of the recently published first consensus statement on the diagnosis and management of pseudohypoparathyroidism-related disorders [7], the diagnosis should be based on clinical and biochemical characteristics, which will vary according to the age of the patient. The diagnosis of AHO should be based on the presence of major criteria (brachydactyly due to premature fusion of the epiphyses and short stature by adulthood) and additional criteria (stocky build, round facies, and ectopic ossifications). Obesity, dental manifestations, and cognitive impairment are present in a subgroup of patients. In the majority of PHP patients, the main clinical manifestations are symptoms of hypocalcemia due to PTH resistance. In patients with PHP1A, resistance to PTH is usually absent at birth and evolves over time (from the neonatal period to 22 years); the first biochemical abnormalities to appear are increased serum PTH and phosphorous levels, whereas hypocalcemia develops gradually 4-5 years later. Other associated endocrine features that support the diagnosis are as follows: early-onset hypothyroidism due to TSH resistance, which is the most common associated endocrine alteration, being present in nearly 100% of PHP1A patients; hypogonadism (due to FSH and LH resistance); hypercalcitoninemia (due to calcitonin resistance); GH deficiency (due to GHRH resistance).

Owing to the important implications for clinical management, early screening for associated conditions and genetic counselling, the molecular cause of PHP should be promptly identified, in order to confirm the diagnosis and characterize the subtype of the disease. Conversely, no genotype-phenotype correlation can be inferred based on mutation analysis [9]. In a large series, the recurrent c.568_571del mutation detected in our patient was found associated with a wide spectrum of phenotypical features. Namely, PTH levels and serum calcium ranged from 57 to 653 pg/ml (reference range 15-55) and from 1.3 to 2.7 mmol/l (reference range 2.1-2.6), respectively [10].

Few studies have focused on the natural history of this disease and on the outcome of its management. The clinical management of hypocalcemia in PHP patients is based on the administration of the active metabolite of vitamin D calcitriol (1,25(OH)_2_ vitamin D) as in the case of our patient, who was previously on therapy with cholecalciferol.

Indeed, owing to PTH resistance, 25OH vitamin D cannot be converted by the renal tubule into its biologically active form, 1,25 (OH)_2_ vitamin D, by PTH-dependent renal 1*α*-hydroxylase. The goal is to maintain normocalcemia and to keep serum PTH levels in the upper portion of the reference range, thereby avoiding PTH suppression, which can be associated with hypercalciuria and renal calcification. On the other hand, long-term and excessive secondary hyperparathyroidism can result in osteitis fibrosa cystica and dislocation of the femoral capital epiphysis (as may have occurred in our patient, who underwent bilateral hip replacement in adult age). PTH, calcium, and phosphorus should be evaluated every six months during treatment in asymptomatic patients, and more frequently when clinically indicated. Patients and families should be instructed with regard to the symptoms of hypo- and hypercalcemia.

The present case underlines the importance of early diagnosis of pseudohypoparathyroidism, as this is crucial to the appropriate treatment and lifelong management of the disease and its complications, which have a great impact on patients and their families. Appropriate genetic counselling is essential in order to establish effective communication with the family, which in turn allows relevant information to be exchanged and genetic testing to be framed within an integrated clinical path. This is in line with the recent consensus statement [7], which recommends a multidisciplinary approach addressing all aspects of the disease and its potential complications.
